# Supplementary material for: Mechanisms governing the pioneering and redistribution capabilities of the non-classical pioneer PU.1
Source: Nat Commun. 2020 Jan 21;11:402. doi: 10.1038/s41467-019-13960-2 (PMC6972792; doi:10.1038/s41467-019-13960-2)
Supplement: Supplementary file 7 — Source data [file 41467_2019_13960_MOESM7_ESM.zip › Source_Data/Figure6/Figure6B_MotifScanOutput/FLI1_PU.1/homerResults/motif14.similar.html]

motif14

## Information for motif14

T
C
G
A
C
T
A
G
T
C
G
A
C
T
G
A
C
T
A
G
C
T
G
A
C
G
T
A
C
T
A
G
C
T
A
G
C
G
T
A
  
Reverse Opposite:  

A
C
G
T
A
G
T
C
A
G
T
C
A
C
G
T
A
G
C
T
A
G
T
C
A
G
C
T
A
G
C
T
G
A
T
C
A
G
C
T
  

|  |  |
| --- | --- |
| p-value: | 1e-115 |
| log p-value: | -2.653e+02 |
| Information Content per bp: | 1.741 |
| Number of Target Sequences with motif | 2267.0 |
| Percentage of Target Sequences with motif | 22.79% |
| Number of Background Sequences with motif | 4789.0 |
| Percentage of Background Sequences with motif | 13.12% |
| Average Position of motif in Targets | 100.5 +/- 51.9bp |
| Average Position of motif in Background | 101.5 +/- 89.3bp |
| Strand Bias (log2 ratio + to - strand density) | 0.1 |
| Multiplicity (# of sites on avg that occur together) | 1.74 |
| Motif File: | file (matrix) reverse opposite |

### Similar de novo motifs found

|  |  |  |  |  |  |  |  |
| --- | --- | --- | --- | --- | --- | --- | --- |
| Rank | Match Score | Redundant Motif | P-value | log P-value | % of Targets | % of Background | Motif file |
| 1 | 0.806 | C A T G T C G A T A C G C T A G C T A G C T A G C T A G G T C A A T C G C T A G C G T A | 1e-69 | -160.657564 | 8.10% | 3.62% | motif file (matrix) |
| 2 | 0.808 | A C T G A C T G T C G A C T A G C T A G C G T A C T A G C T A G C G T A | 1e-52 | -121.923944 | 6.45% | 2.95% | motif file (matrix) |
| 3 | 0.767 | A C T G T C G A A C T G C T A G C G T A A C T G A C T G C G T A | 1e-52 | -121.007845 | 12.40% | 7.37% | motif file (matrix) |
| 4 | 0.753 | G C T A A T C G G T C A C G T A A C T G G T C A G C T A C A T G A C T G C G T A C T A G A T C G G C T A | 1e-40 | -93.493902 | 2.79% | 0.90% | motif file (matrix) |
